# 3D mouse pose from single-view video and a new dataset

**DOI:** 10.1038/s41598-023-40738-w

**Published:** 2023-08-21

**Authors:** Bo Hu, Bryan Seybold, Shan Yang, Avneesh Sud, Yi Liu, Karla Barron, Paulyn Cha, Marcelo Cosino, Ellie Karlsson, Janessa Kite, Ganesh Kolumam, Joseph Preciado, José Zavala-Solorio, Chunlian Zhang, Xiaomeng Zhang, Martin Voorbach, Ann E. Tovcimak, J. Graham Ruby, David A. Ross

**Affiliations:** 1 Google, 1600 Amphitheatre Parkway, Mountain View, CA 94043 USA; 2 Calico Life Sciences LLC, 1170 Veterans Blvd., South San Francisco, CA 94080 USA; 3 Translational Imaging, Neuroscience Discovery, Abbvie, 1 N. Waukegan Rd., North Chicago, IL 60064-1802 USA

**Keywords:** Medical research, Computer science, Scientific data, Imaging, Software, Computational models

## Abstract

We present a method to infer the 3D pose of mice, including the limbs and feet, from monocular videos. Many human clinical conditions and their corresponding animal models result in abnormal motion, and accurately measuring 3D motion at scale offers insights into health. The 3D poses improve classification of health-related attributes over 2D representations. The inferred poses are accurate enough to estimate stride length even when the feet are mostly occluded. This method could be applied as part of a continuous monitoring system to non-invasively measure animal health, as demonstrated by its use in successfully classifying animals based on age and genotype. We introduce the Mouse Pose Analysis Dataset, the first large scale video dataset of lab mice in their home cage with ground truth keypoint and behavior labels. The dataset also contains high resolution mouse CT scans, which we use to build the shape models for 3D pose reconstruction.

## Introduction

Many human clinical conditions and the corresponding animal models result in abnormal motion^1^. Measuring motion is a requisite step in studying the health of these subjects. For animal subjects, researchers typically conduct measurements manually at high cost, limited resolution, and high stress for the animals. In this work, we present a low-cost, non-invasive, computer-vision based approach for continuously measuring the motion as 3D pose of laboratory mice.

To study animal models of movement disorders, such as Parkinson’s disease or tremor, or even generally measure behavior, researchers rely on manual tools such as the rotarod, static horizontal bar, open field tests, or human scoring^2,3^. Increasingly complex automated tools to study gait and locomotion are being developed^4,5^. Computer vision and machine learning are creating new measurement opportunities in home cage environments for 2D tracking or behavior^6–12^. Whereas open fields are arenas without features, a home cage is an enclosure furnished with familiar bedding, food and water, as well as enrichment items that allow the animals to exhibit a wide range of movements and behaviors. So far, only a few studies measure 3D motion in home cages at all, and only at coarse resolution or number of joints or requiring multiple cameras^13–17^. Nevertheless, these new measurement tools are offering compelling opportunities for new analyses^13,17–19^.

In parallel, computer vision and machine learning are leading to great improvements in determining human 3D pose from images. Models for optimizing a kinematic model to fit image data^20^ are being paired with improvements in estimating 2D poses^21–23^. By combining these methods with libraries of human shapes^24^ and human poses, 3D human pose estimates can be grounded to real kinematic models and realistic motions^25–27^. Ongoing research is improving the spatial and temporal coherence^28–30^.

This work adapts these techniques originally developed to infer 3D human pose to mice. We predict 2D keypoints for mice then optimize for the 3D pose subject to priors learned from data. To infer human poses, databases of human shapes, poses, 2D keypoints, and 3D keypoints are readily available, but none of these are available for mice. The lack of data presented unique challenges to accurately infer 3D poses. We overcome these challenges by collecting new data and adapting where needed. We design our algorithms and collect data to achieve two goals.

**Figure 1:**
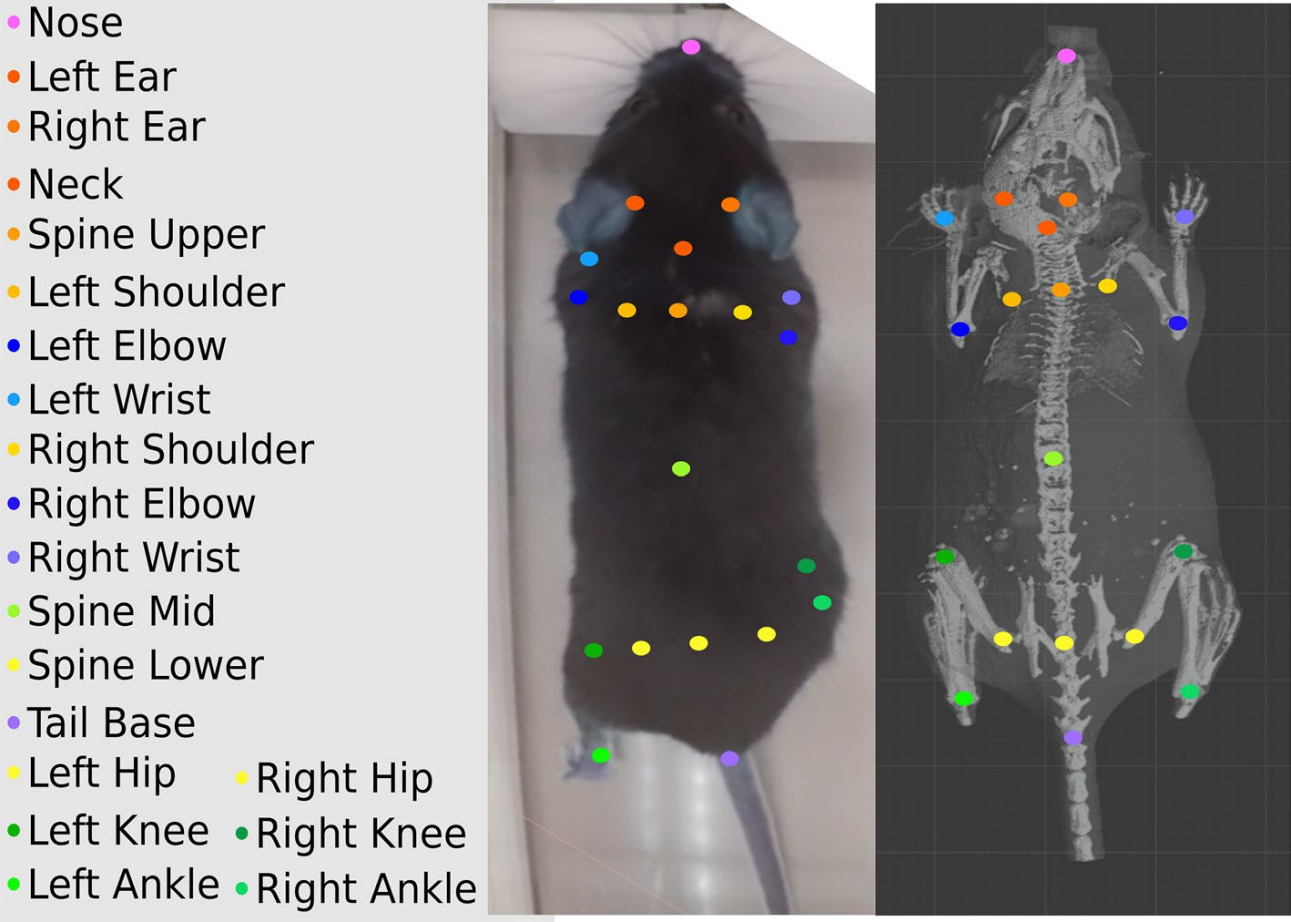
*Left:* The 2D keypoint names and corresponding color-coded markers shown in the labeling interface. *Center:* A labeled image of a mouse with the keypoint legends to the left. *Right:* The high resolution CT scan segmented for bone in light colors, and segmented for the skin in darker colors with the corresponding keypoint locations at a neutral pose.

- Scalability. The algorithms are able to monitor mice in their home cage continuously for prolonged duration, and can do so over a large number of cages at the same time. Although the open field assay is one of the most commonly used assays in research, it induces stress to the animal and variance to the study outcome. Home cages provide subjects the most natural settings and facilitate unbiased physiological and behavior studies^31^. Measurements of activities in a multitude of home cages pose fresh challenges^15^, and call for robust algorithms.
- Robustness. Occlusion, both from the animal itself and the objects in the cage, is the main obstacle to reconstructing the pose accurately. We approach the problem by employing a full set of anatomically significant keypoints (Fig. 1). We have observed that the model trained with more keypoints generalizes with occluded body parts. Compared with the 20 keypoints we use in our data, other large scale datasets provide fewer keypoints. For example, the CalMS21 dataset^32^ has 7 keypoints, the MARS dataset^33^ has 9, and the PAIR-R24M dataset^34^ has 12. The Rat 7M dataset^35^, although capturing 20 markers, has less than 16 keypoints on the animal body.

To support reproducibility and encourage future research, we make our annotated training and evaluation data, and the pose reconstruction models and code publicly available. The Mouse Pose Analysis Dataset released here has the following features: 3D high resolution CT scans of mice of with a wide weight distribution and both sexes; over 400 video clips of mouse activities in their home cage, both in light and dark cycles; 20 keypoint labels on each mouse and 7 behavior labels; 3D ground truth keypoint labels from a 3D capture rig with multiple cameras and a Kinect device.

We validate our method by demonstrating the metric accuracy of the inferred 3D poses, the predictive accuracy of health related attributes, and the correlation with direct measurements of gait. In each case, the inferred 3D poses are useful, detailed measurements.

The study is reported in accordance with ARRIVE guidelines (https://arriveguidelines.org).

## Related work

### 2D pose estimation

The development of deep learning based animal pose estimation is deeply influenced by human pose algorithms (see^36–39^ for recent surveys.) DeepLabCut^40^ employs transfer learning and achieves human accuracy with a small number of labeled samples and spurred many further developments. LEAP^41^ speeds up the annotation process even more by iteratively fine tuning the model and providing initial guesses on new training samples. DeepPoseKit^42^ eliminates the preprocessing step in LEAP and claims to increase the robustness over factors such as rotation and lighting changes. All three methods work in open field settings; however, it is not clear how they perform with home cage images. Another line of improvement is to utilize spatio-temporal consistency between adjacent video frames. OptiFlex^43^ computes optical flow information from the keypoint heat maps generated from a base model, and shows improvement in accuracy and robustness. OpenPifPaf^44^ uses Composite Fields, including intensity, association and temporal association fields, to detect and track keypoints. Instead of adding these Composite Fields at the end of the network, DeepGraphPose^45^ encodes the spatio-temporal structure in a graphical model. The advantage of such a model is the ability to infer occluded keypoints.

### 3D pose estimation

While 2D pose is sufficient for many biological questions, 3D movement and kinematics are indispensable in understanding the connections between neural and motor systems.

3D pose can be obtain by triangulating 2D keypoints with multiple cameras^46–48^, and/or using depth sensors^49–52^. We construct a multi-view 3D capture rig, which includes a Kinect device, (detailed in “Multiview 3D pose reconstruction” Section) to evaluate our single view 3D reconstruction algorithm. The added complexity limits the scalability of such systems, so it is not feasible to install the extra devices to monitor more than a dozen cages. Recent advances in machine learning has seen methods that reconstruct 3D pose from single camera views. LiftPose3D^53^ estimates 3D joint location from single views by training a network (the *lift* function) on 3-D ground truth data. The training data is augmented with different camera angles and bone lengths, which enables the network to solve camera parameters implicitly and cope with variations in animal sizes. In comparison, we estimate camera parameters and build the shape distribution explicitly. Dunn et al.^13^ regresses a volumetric representation of the animal, from which 3D pose is calculated.

Different from these end-to-end learning algorithms, we cast the 3D pose estimation as an optimization problem with a mouse skeleton model^54^. By encoding the 3D joint angles explicitly, the model outputs are readily interpretable. More importantly, the 3D skeleton model imposes a strong prior (see “Kinematic chain and 3D pose prediction” Section), which both overcomes missing observations from occlusions and serves as a regularization on the over-parameterized joint space.

## The mouse pose analysis dataset

The Mouse Pose Analysis Dataset includes 455 video clips of C57BL/6N and Diversity Outbred mice and CT images of 80 C57BL/6N mice. The goal is to support diverse research problems in animal physiology and behavior by providing a dataset that covers lab mice of typical genotypes, sexes, weight and activities in their home cages.

All CT studies were performed in compliance with AbbVie’s Institutional Animal Care and Use Committee and the National Institute of Health Guide for Care and Use of Laboratory Animals Guidelines in a facility accredited by the Association for the Assessment and Accreditation of Laboratory Animal Care.

All video-capture-related research was performed as part of Calico Life Sciences LLC AAALAC-accredited animal care and use program. All research and animal use in this study was approved by the Calico Institutional Animal Care and Use Committee (IACUC).

### Data collection

#### CT Scans

Male and female wild-type C57BL/6N mice were obtained from Charles Rivers Labs (Wilmington, MA). Animals were acclimated to the animal facilities for a period of approximately one week prior to commencement of experiments. Animals were tested in the light phase of a 12-h light/12-h dark schedule. Anesthesia was induced using isoflurane. Isoflurane levels were maintained between 1 and 2.5 vol% in oxygen. The data was acquired using a Siemens Inveon microPET/CT (Knoxville, TN). Animals underwent CT scans with the following settings: Total rotation of $220^\circ$ with $1^\circ$ steps after 20 dark/light calibrations. The transaxial and axial field of view were 58.44 and 92.04 mm respectively. Exposure time was 800 ms with a binning factor of 2, the effective pixel size was 45.65 $\upmu$m. The Voltage and current settings were 80 kV and 500 $\upmu$A respectively. Total scan time per animal was estimated as 1010 s. CT images used the common cone-beam reconstruction method, included Houndsfield unit calibration, bilinear interpolation and a Hamming reconstruction filter. Reconstructed CT images were converted to DICOM using VivoQuant software (InVicro, A Konica Minolta Company).

#### Video frames

Diversity Outbred (J:DO) mice were obtained from The Jackson Laboratory (Strain #009376; Bar Harbor, ME). C57BL/6N were obtained from Charles Rivers Labs (Wilmington, MA).

To build a general purpose visual pipeline, we acquired video of a Diversity Outbred strain of mice that have a range of weights (approximately 20–60 g), sexes (female or male), ages (1–3 years), and coat colors (albino, black, agouti). The mice were placed in monitoring cages each outfitted with a single camera (Vium). During this time, mice were housed singly and provided with running wheels and nesting enrichment (cotton nestlets). Each video was recorded at 24 frames per second. During the dark cycle, infrared illumination was used. From this diverse collection of videos, we manually selected 455 video clips where the animals perform one of the following behaviors: standing, drinking, eating, grooming, sleeping, walking or running on the wheel. Since most activities happen in the dark cycles, majority (96%) of the clips are infrared images. Each clip is 0.5 s long and sampled at 24 HZ. Activities were manually labeled by the researchers by watching the clip and surrounding context. Another distinct subset of 310 clips were manually selected for diverse poses by the researchers. The 2D pose of the mouse in each of 12 frames from each clip were annotated by trained animal technicians yielding 3720 annotated frames. The pose annotation pipeline is described in “Keypoints and behavior annotation” Section. As we hope these data sets are useful for the community to train and evaluate similar systems, we release the pose and behavior annotations along with the corresponding frames.

We collected three further sets of experimental video data used only for evaluation: Continuous, Multiview, and Gait. The *Continuous* video data is 14 days from 32 cages. Eight animals are 1-year old, homozygous Eif2b5R191H/R191H knockout mice on a C57BL/6N background^55^; eight are 1-year old, heterozygous knockout controls; eight are 1-year old, C57BL/6N mice; and eight are 2-months old, C57BL/6N mice. The knockout mice have a deletion that causes motor deficits^55–57^. The knockout mice and heterozygous controls are littermates on a C57BL/6N background, but have been inbred for several generations. Each mouse has three attributes: age (either 12 or 3 months old), knockout (either a full knockout or not), and background (either a littermate with a knockout or a C57BL/6N). The *Multiview* video data is 35 consecutive multiview frames of a single C57BL/6N mouse in a custom capture rig (described below). Note that the depth information from the Kinect sensor is too noisy to use as ground truth by itself. Instead, we only use the RGB values in the multiple-view set up. The *Gait* video data is of a single C57BL/6N mouse walking on a treadmill with cameras installed below with corresponding commercial analysis tools (DigiGait) with an additional camera mounted above (GoPro) that we use for analysis. The Multiview and Gait video data was captured at 30 frames per second. These experimental video sets are only used for demonstrating the utility of our method and will not be released. All experiments are approved by an Institutional Animal Care and Use Committee.

It is worth noting that there is a large body of literature on speed and frequency of mouse locomotion. Though the stride length and frequency are dependent on the speed, it has been observed in multiple studies that the stride frequency falls between 3 and 10 HZ^58–60^, which means the Nyquist rate of typical mouse movements is under 24 HZ. A 24 HZ camera is therefore sufficient to record many behaviors including locomotion, but for some faster motions beyond the scope of this study (e.g. whisker dynamics), a faster camera could be used. The algorithms do not depend on the camera frame rate.

### Keypoints and behavior annotation

Ten scientists and technicians participated in the keypoint and behavior annotation. They were asked to view the video clips and label the clips from the 7 behavior labels (see Table  1 for the list). They were instructed to draw a bounding box around the animal and to label keypoints corresponding to 3D skeleton joints (Fig.1). Non-joint keypoints are defined as follows. The Lower Spine point is at the mid point between the two hip joints and on the spine. The Upper Spine is similarly defined between the two shoulder joints. The Middle Spine is half way between the Upper and Lower spine points on the spine. The annotators were asked to mark their best guess when keypoints are occluded. The objective was to obtain possibly noisy labels from experts rather than no labels at all.

### Dataset statistics

The CT images include mice of different ages and weights. Mice were grouped based on weights and sex, with 10 per group. Group 1 females weighed $15.7 \pm 0.74$ g and males weighed $18.4 \pm 0.98$ g. Group 2 females weighed $24.9 \pm 1.8$ g and males weighed $23.2 \pm 1.36$ g. Group 3 females weighed $28.0 \pm 2.52$ g and males weighed 27.3 ± 0.97g. Group 4 females weighed $35.3 \pm 6.11$ g and males weighed $38.7 \pm 3.00$ g.

**Figure 2:**
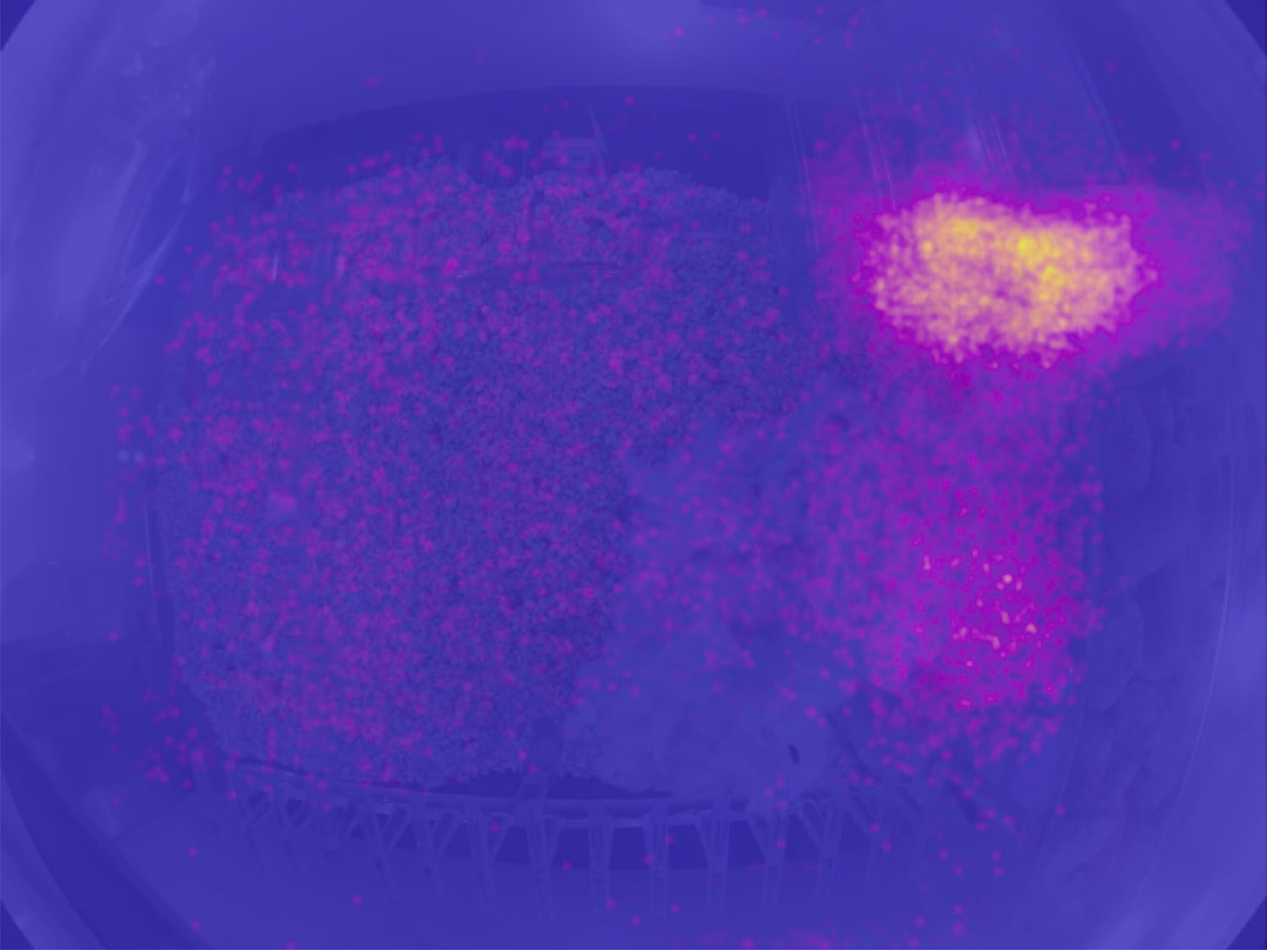
A heatmap of all annotated mouse keypoints displayed in the home cage. Each dot represents one keypoint. Majority of the activities happen on the wheel and near the feeder.

The video frames consist of 39% C57BL/6N subjects and the rest Diversity Outbred. Table 1 shows the distribution of behavior labels among the video frames. Figure 2 shows the aggregated locations of the mice. Given the nocturnal nature of mice, most video frames (96%) are from the night cycle. Since we emphasize pose analysis during mouse movement, over half of the annotations are mouse running on wheels.

**Table 1:** Percentage of human-labeled mouse behavior of the video frames.

Behavior	Percentage
Drinking	1.8
Eating	9.9
Grooming	16.5
Sleeping	4.4
Standing	5.5
Walking	11.1
Wheel	50.8

### Data availability

The data used for training and evaluating the 2D and 3D pose estimation are released as part of this publication. The data for demonstrating the utility on some biologically relevant tasks will not be released because it is specific to this paper and larger than what is easily shareable. We do not believe this limits the ability to reproduce our method or evaluate the performance for 2D and 3D pose estimation. Specifically, we release the 5460 annotated frames from 455 videos annotated for training and evaluating 2D pose and the 80 CT-scans used to construct the shape prior. You can request access to the data via this link: https://google.github.io/mouse-pose-analysis-dataset/.

### Related datasets

There are a few mouse and rat datasets of comparable size publicly available. The MIT Mouse Behavior Dataset^61^ contains 10.6 h of continuously labeled side-view video (8 day videos and 4 night videos) for the eight behaviors of interest: drink, eat, groom, hang, micro-movement, rear, rest, walk. The mice are singly housed in their home cage. There are no keypoint labels.

The Caltech Mouse Social Interactions (CalMS21) Dataset^32^ consists of 6 million frames of unlabeled tracked poses of interacting mice in home cages, as well as over 1 million frames with tracked poses and corresponding frame-level behavior annotations. Seven keypoints (the nose, ears, base of neck, hips, and tail) are labeled.

The Rat 7M Dataset^35^ contains 10.8 h of videos across 6 different rats and 30 camera views, totaling about 7 million frames, across a wide range of rat poses. The frames are captured from 20 markers attached to the animals using an array of cameras.

The PAIR-R24M Dataset^34^ contains 24.3 million frames of RGB video and 3D ground-truth motion capture of dyadic interactions in laboratory rats from 18 distinct pairs of rats and 24 different viewpoints. Each frame provides the 3D positions of 12 body landmarks and is associated with one of 11 behavioral categories and 3 inter-animal interaction categories.

The first two datasets have few or no labeled keypoints. While the latter two have more labeled keypoints, they contain open field images rather than home cage images. The Mouse Pose Analysis Dataset is the first large scale dataset of lab mice in their home cage with full set of keypoint and behavior annotations.

## Methods

### Mouse pose prediction

**Figure 3:**
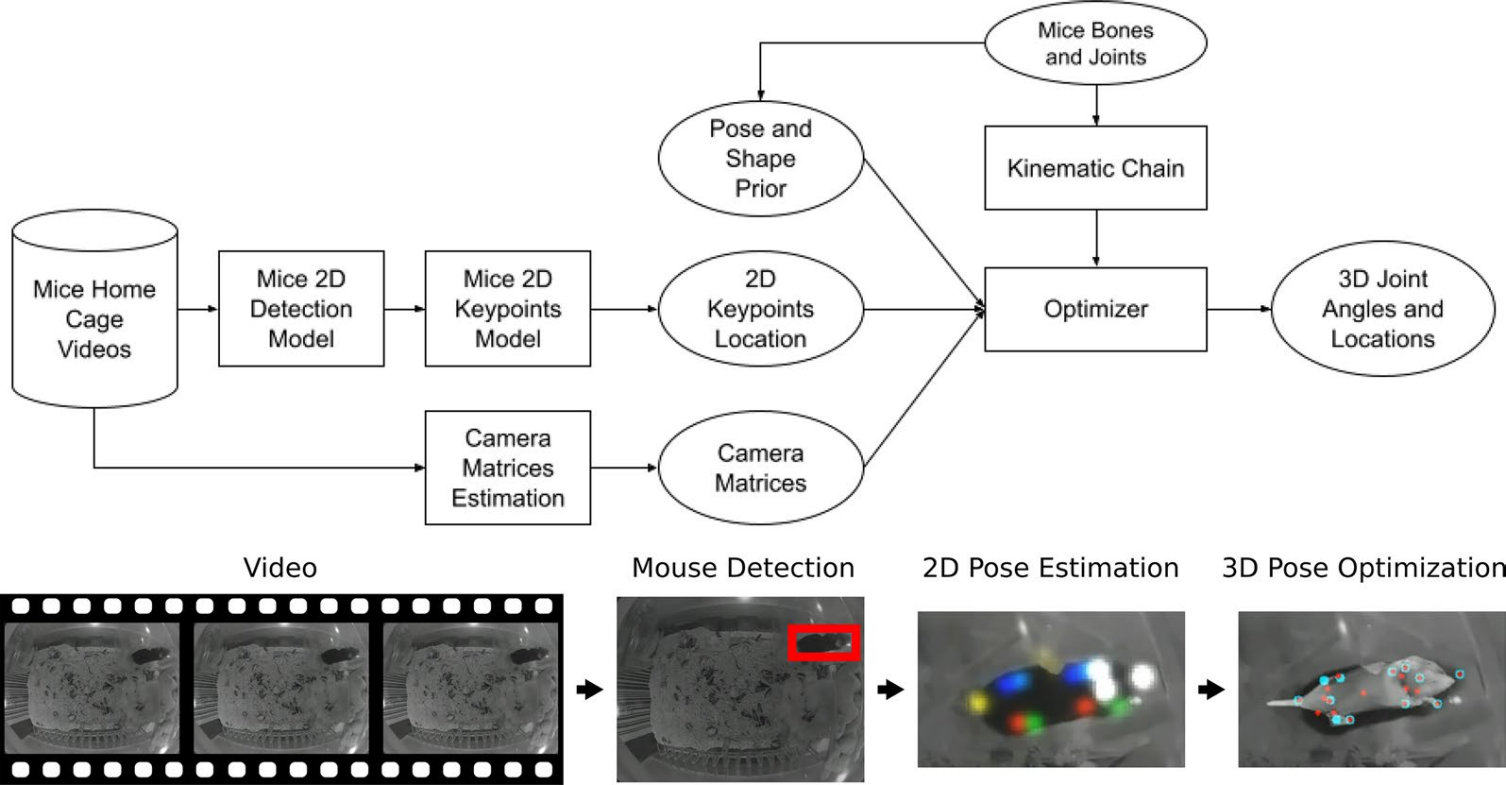
*Top*: Pipeline diagram. Rectangular boxes are algorithms and processes. Ellipses are intermediate and final results of the pipeline. *Bottom*:Pictorial depiction of the pipeline. It operates over frames of a video (left panel). For each frame we run a 2D object detector trained to detect mice (second panel, box indicating a detection). We apply a 2D pose model to detect mouse keypoints at the detected location (third panel, colored heatmap indicating joint locations with arbitrary colors). Finally, we optimize for the 3D pose of the mouse (right panel, blue points are peaks of the keypoint heatmaps in previous stage, red points are projected 3D keypoints from the optimized pose, grey 3D mesh overlaid on the image).

Our feature extraction pipeline (shown in Fig. 3) includes three stages: bounding box detection, 2D pose prediction, and 3D pose optimization. These stages have been shown to be effective for human 3D pose estimation^25,62,63^. We release the machine learning models and the code of the pipeline at https://github.com/google/mouse-pose-analysis.

#### 2D detection and pose prediction

We adapt a Single-Shot Detector^64^ to detect the mouse and a Stacked Hourglass Network^22^ to infer the mouse’s 2D pose, similar to other work adapting human pose models to laboratory animals^9,11^.

The detection and pose models both require training data, which we generate by labeling 20 joint positions along the body, and take the minimal box encompassing all points to be the bounding box. Models are pretrained on COCO^65^ and the prediction heads for human keypoints are replaced with those for mouse keypoints. For the Continuous video data, we label 3670 images for the training set and 628 for the test set. For the Gait video data, we fine-tune the Continuous video model on an additional 329 labeled image training set and test on 106 images. Frames are selected manually and then annotated to cover the diversity of input images across cages and times.

We evaluate our pose model with the Object Keypoint Similarity (OKS) score used on COCO^65^: $\sum _{i}\exp (-\textbf{d}_i^2 / (2\textbf{k}_i^2\textbf{s}^2)) / 20$, where $\textbf{d}_i$ is the Euclidean distance between the prediction and ground truth, $\textbf{s}$ is the object scale as the square root of the bounding box area, and the per-keypoint falloff, $k_i$, is set to the human median of 0.08 for all keypoints (See http://cocodataset.org/#keypoints-evalforfurtherOKSdetails). This setting is equivalent to measuring the proportion of predicted keypoints with a certain radius of the ground truth point proportional to the bounding box size. The radius decreases, requiring more accurate predictions, for higher OKS thresholds and smaller bounding box sizes. Accuracy is computed as the percentage of predicted keypoints greater than a threshold OKS score/pixel radius in Table 2.

**Table 2:** The 2D pose accuracy as proportion of keypoints with OKS scores above the specified thresholds, T, for different joints across different data sets. R is the radius (in pixels) of the region corresponding to the threshold for the average bounding box size.

T	R	Nose	Shoulder	Hip	Wrist	Ankle
		Continuous video test dataset—home cage				
0.5	11.4	0.92	0.96	0.93	0.91	0.91
0.7	8.2	0.87	0.93	0.85	0.77	0.75
0.9	4.4	0.72	0.64	0.47	0.44	0.34
		Gait video test dataset—gopro over digigait				
0.5	25.6	1.	0.96	0.89	0.6	0.80
0.7	18.4	0.99	0.79	0.70	0.24	0.67
0.9	10.0	0.70	0.41	0.23	0.09	0.26

#### Kinematic chain and 3D pose prediction

We adapt the human 3D pose optimization strategy from^20^ to mice because similar optimization strategies are successful with inferred 2D poses and relatively little 3D ground truth data^25^.

The 3D pose is defined on a kinematic chain, consisting 18 out of the 20 joints in Fig. 1 (the ears are excluded). All joints are modeled as spherical, leading to 54 total number of joint angles.

Since the camera and the lens are fixed to each cage, we pre-calibrate the intrinsic and extrinsic parameters, which are available on the dataset website. We iteratively update the 3D joint angles $\textbf{a}$ and bone lengths $\textbf{l}$ on the kinematic chain, represented by $T(\textbf{a}, \textbf{l})$, to minimize the distance between the input 2D keypoint locations and the projected 3D joint locations (Eq. 1).

1 $$\begin{aligned} E(\textbf{a}, \textbf{l})=\Sigma _i\Vert \textrm{proj}(T(\textbf{a}, \textbf{l})) - k_i)\Vert ^2+\lambda _p p_p(\textbf{a})+\lambda _s p_s(\textbf{l}) \end{aligned}$$

We improve the stability and convergence of the 3D pose optimization by using the shape prior $p_s$ and the pose prior $p_p$. The priors are constructed similar to the SMPL model^25^. We build the pose prior from a multiple-view reconstruction of the 3D pose (see below), augmented with hand-posed models, which have joint angles set in a 3D modeling software to match the apparent mouse pose in a set of images that cover poses that may not appear in the multiple-view videos. From these 3D poses, we align and scale the poses so that the vector from the base of the neck to the middle of the spine is defined as the x-axis and unit length, and then we fit a Gaussian mixture model with 5 components to the data. $\lambda _p$ was set to a small value so that the pose prior had a weak effect similar to keeping the feet towards the ground, but not constraining the recovered poses to the small mixture distribution.

To build the shape prior, we collect all the bone lengths from the CT scans in the dataset, which covers mice of different gender, age and weight. We fit a 7-component Gaussian mixture model to the lengths to form the shape prior.

The optimization is over-parameterized where the overall size and the distance to the camera are confounded, which can result in arbitrary scale and physically implausible rotations. We solve the complication by constraining the animal to a fixed distance from the camera. Similar scene constraints are a common approach to reconstructing physically meaningful 3D poses^28,30^.

### Multiview 3D pose reconstruction

To generate ground truth 3D pose data for validation and constructing a pose prior, we build a custom, multiview 3D capture rig. A top-down RGB+Depth camera (Kinect) and two side RGB cameras with synchronized timing are calibrated with overlapping fields of view of a mouse cage. We label the 2D joint positions in synchronized frames from each field of view and triangulate the 3D location of each joint position that minimizes the reprojection errors. The multiview reconstructions are used to evaluate the single-view reconstruction quality. A separate and larger set is used to construct the pose prior.

### Biological attribute prediction

#### Mouse description

The Eif2b5R191H/R191H knock-in mutant mouse model used in the study is generated in the background strain C57BL/6J^55^. Eif2b mutants are known to have motor defects such as increased slips on a balance beam, decreased inverted grid hanging time, decreased rotarod duration, and a different stride^55–57^. In this study, we compared R191H homozygous mutants (KO) to their heterozygous litermates (HET) to demonstrate we can detect locomotor deficits in a known mouse model to their genetically similar siblings. Mice were measured at 3 months and 12 months. We also measured a set of C57BL/6J mice (WT) and compare to the HET group at the same age. HET mice were not backcrossed a sufficient number of times to control for genetic drift. As a result, comparisons between the HET and WT groups cannot distinguish differences between drift and mutation-caused phenotypes, but any observed differences point to the sensitivity of our method.

#### Attribute prediction

To assess which representations preserve information about motion dynamics, we train a black-box artificial neural network model to predict biological attributes in the Continuous video data. Because we want to study gait and not other factors, we limit the analysis to sequences when the animal is on or near the wheel during the night cycle, when the mice are more active. We train on and predict labels for 10 s intervals, but evaluate performance across the aggregated prediction scores for each animal to normalize for the amount of time on the wheel. Data are split into the training (63057 segments) and test (32163 segments) sets with disjoint sets of mice in each. For each data representation we test, we train a convolutional neural network with kernel size 24 to predict each label independently. We trained the models using the Adam optimizer^66^ with a sum of binary cross-entropy losses per attribute for 5 epochs. We perform a hyperparameter sweep over the number of layers in the network [2, 3, or 4], the number of hidden units in each layer [32, 64, 128, 256], and the learning rate [0.0001, 0.00001, 0.000001] using half the training set for validation. We report the best accuracy for each representation on the test set.

### Gait measurements

Direct measurements of gait parameters are obtained via a commercial system (DigiGait). We use the aggregated stride length from the Posture Plot report as well as the individual stride length measurements from the commercial system. We calculate similar measurements from our method by computing the duration of strides from the reconstructed pose and multiplying by the known treadmill speed to calculate the stride length. The aggregate duration of the stride is calculated as the wavelength of the Fourier spectrum peak magnitude and the individual stride durations are calculated as peak-to-peak times.

## Results

### Inferred 3D poses

**Figure 4:**
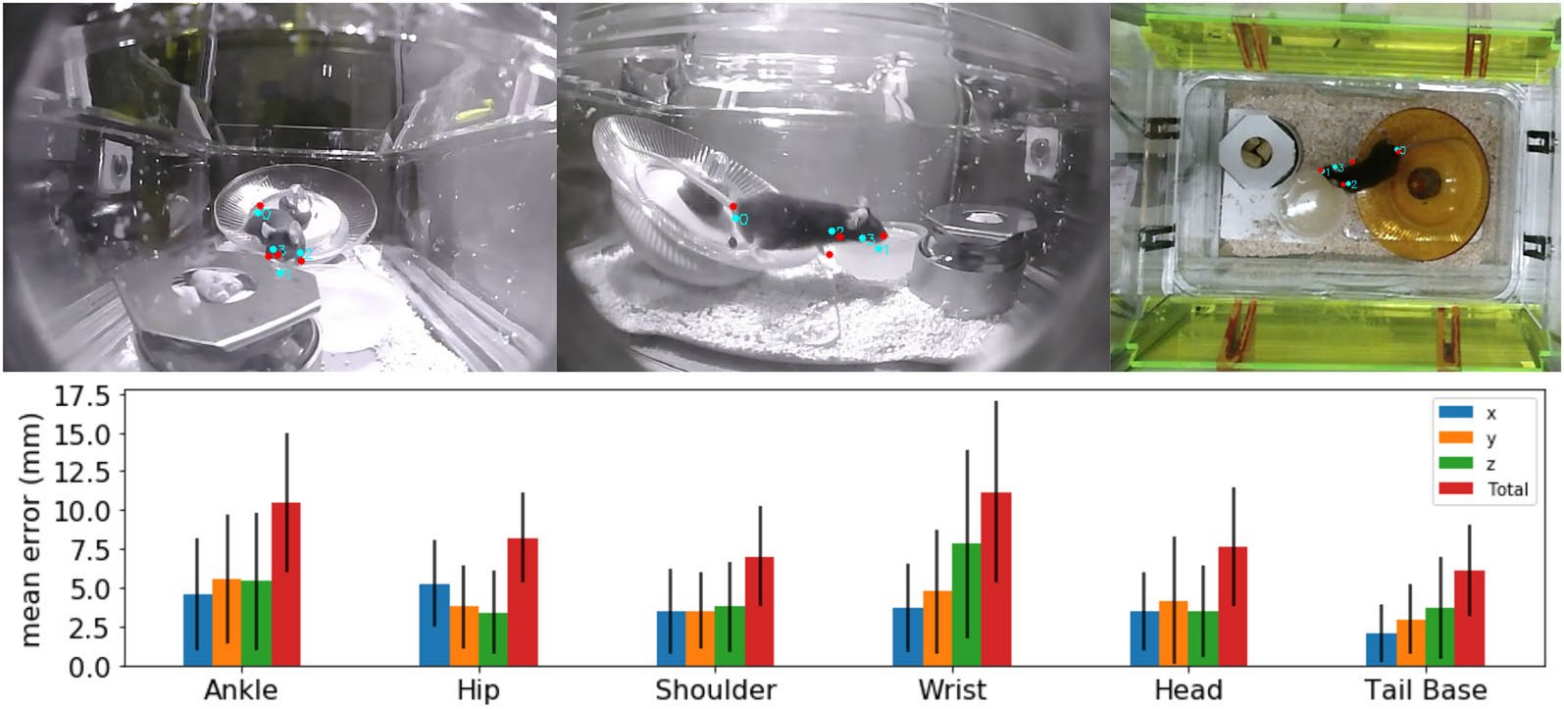
Comparison of multi-view and single-view reconstructions. The error bars are $\pm 1$ SE. The top three panels show three views of the mouse at the same time point. Red dots are reconstructions from triangulation and cyan dots from our single-view reconstruction. Four of 20 joints are shown as examples (0: tail, 1: noise, 2: left paw and 3: right paw).

We quantitatively evaluate the quality of our 3D poses on the Multiview video data set. After determining the ground truth 3D pose from multiple views (see “Methods” Section), we calculate how well we reconstruct the pose from the top down view alone. The inferred 3D pose is registered to the ground truth pose and we quantify the error in the inferred 3D pose in millimeters in Fig. 4, which shows the RMSE of 35 measurements per joint. The error bars are 1 standard error. The errors on tail, shoulder and head are smaller than those of ankle, hip and wrist, whose 2D poses are noisier due to occlusion. The average error for each joint is less than 10 mm. As the average body length of mice is approximately 10 cm, this represents less than 10% relative error. We cannot find another monocular 3D pose reference that lists numbers to compare against. Although these numbers allow room for improvement, we demonstrate further results that this accuracy is sufficient to enable health predictions and extraction of gait parameters.

### Biological attribute prediction with 3D pose

After inferring the 3D poses, we show that the extracted representations are sufficient to infer subtle differences in age, genetic background, and heterozygous versus homozygous knockouts. We use Continuous video data attributes to assess how easily models can predict biological attributes from different features: the 2D bounding box, the 2D keypoints, the 3D keypoints, and the 3D joint angles. We train a range of artificial neural networks on each representation and present the best results for each feature on a held out set of 16 animals in Table 3. Of these, the 3D joint angles outperform the others by being able to perfectly classify each animal in the test set, while the others make one to three mistakes on the 16 test set animals.

**Table 3:** Table of the classification accuracy ($mean \pm standard$ error of 5 training runs) for each input representation on a held-out set of animals for three attributes: whether the animal is 12 or 3 months old (Age), whether the animals is a litter mate of a knockout (Bkgrd), and whether the animal is a knockout (KO).

Feature	acc (Age)	acc (Bkgrd)	acc (KO)
2D boxes	$0.86 \pm 0.04$	$0.82 \pm 0.01$	$0.89 \pm 0.03$
2D points	$0.85 \pm 0.02$	$0.81 \pm 0.02$	$0.91 \pm 0.02$
3D points	$0.88 \pm 0.00$	$0.82 \pm 0.02$	$0.90 \pm 0.03$
3D angles	**1.00 ** ± **0.00**	**1.00** ± **0.00**	**1.00 **± **0.00**

Best result in each column is in bold.

### Accurate gait measurements from 3D pose

**Figure 5:**
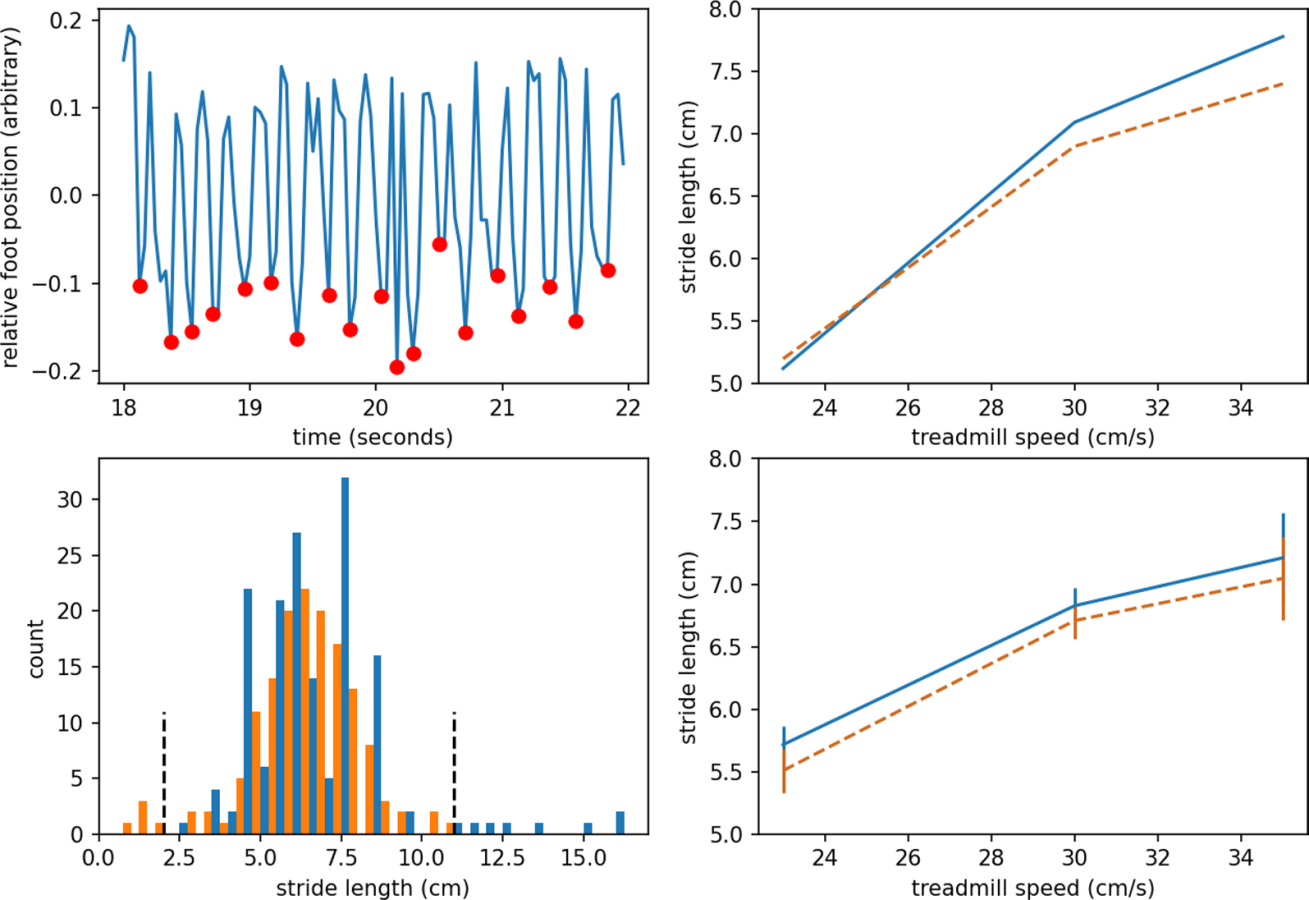
*Top left:* An example time series of the foot position in arbitrary units. The periodic structure of gait is clearly visible. Red dots indicate peaks used in computing the stride length. *Top right:* The peak frequency in the foot position reconstruction $\times$ belt speed (blue, solid) and DigiGait posture plot stride length (orange, dashed). *Bottom left:* The distribution of stride lengths from the pose reconstruction (dark blue) and DigiGait (light orange). Dashed, black, vertical lines indicate outlier thresholds for statistical modeling. *Bottom right:* Stride lengths by treadmill speed for reconstructed pose (blue, solid) and DigiGait (orange, dashed). Error bars indicate ±1 SEM.

To further validate our method, we compare the measurements of strides by our system with the the measurements from a DigiGait system that directly images the feet from below. We infer the 3D poses as viewed from above using our method, estimate the strides and compare the output to the direct stride measurements by the DigiGait system in Fig. 5. We find that we can recapitulate multiple direct measurements.

The stride length estimated from the magnitude of the Fourier spectrum of the foot position over several seconds matches the aggregated Posture Plot stride length very well. Because the spectrum analysis aggregates over time, it should be more accurate than single stride analyses and avoids sampling noise due to the limited frame rate we use (24 fps). However, we cannot compute statistics from an aggregated number, so we also compared noisier individual stride estimates.

We measure the peak-to-peak times to estimate the individual stride lengths and compare the distribution to the direct measurements. Excluding 13 asymmetric outliers beyond 2.3 $\sigma$ from the mean, the measurements from our system were not significantly different from the direct measurements (2-way ANOVA, main effect of measurement system: df = 289, t$=-$ 0.8, $p=0.424$). While statistics cannot prove distributions are identical, we can claim that our measurements are similar to the commercial system except that DigiGait outliers are short strides while ours are long strides.

### Behavior classification

**Figure 6:**
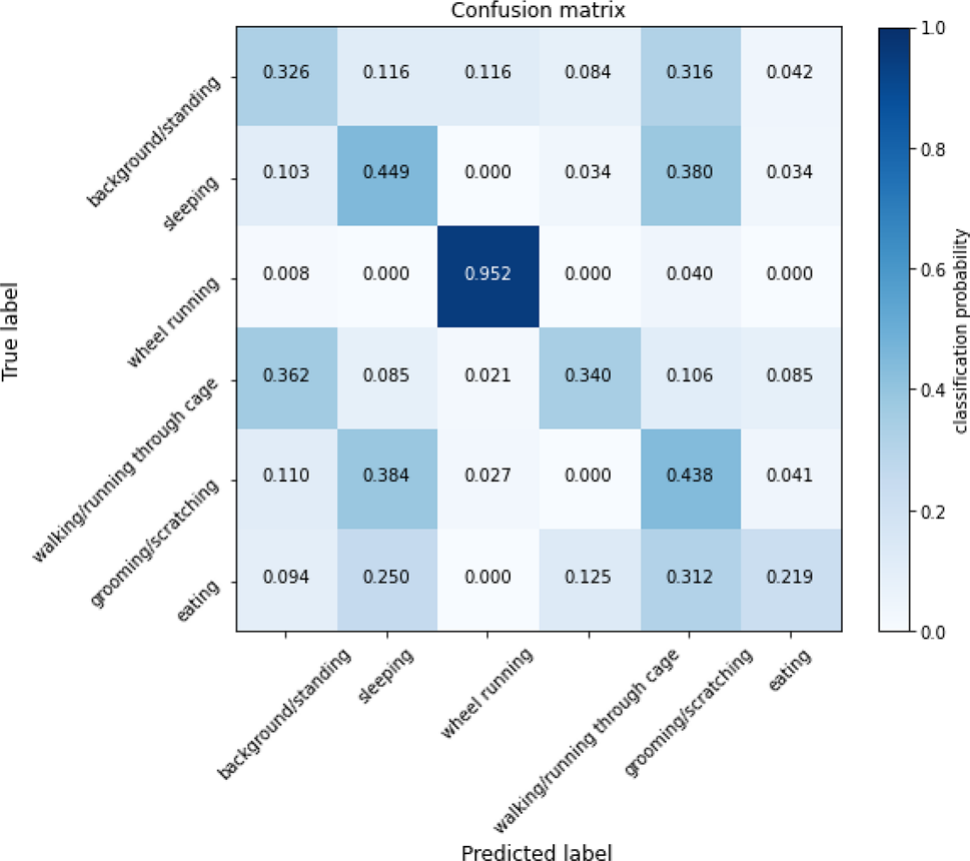
Representative confusion matrix for behavior classification. Each row represents the predicted classification for a given true positive label. Each column is a different output prediction. This particular confusion matrix is for the Images model, but the pattern is consistent across input types.

We learn and evaluate inferring the behavior of mice on manually labeled set of 1254 training videos, 400 validation videos, and 400 test videos. We intentionally use a small data set to mimic the common need in biological research to reuse components to solve new tasks with limited labeled data available. As behavior can often be inferred from a single frame, we compare against an convolutional neural network in addition to low-dimensional extracted features. We extract ResNet embeddings for 12 consecutive frames, average the features over time, and predict the behavior with a 2-layer MLP. We used convolutional networks as described in “Biological attribute prediction” Section to infer behavior from the low-dimensional extracted features. We trained with the Adam optimizer for 25 epochs. We find in Table 4 that the bounding box outputs of our pose pipeline can infer the behavior better than adapting a deep convolutional neural network. The 2D and 3D keypoint representations also do nearly as well. The models most often confuse classes with similar poses, but different amounts of motion, such as classifying “walking/running through the cage” as “standing/background” or “sleeping” as “scratching/grooming” as seen in Fig. 6. One hypothesis is that restricting the input to just the bounding box locations helps the model avoid over-fitting on irrelevant details and better detect small changes in position. A benefit of using our method is that different stages of the pipeline offer different levels of granularity and avoid the computational cost of running multiple convolutional or other expensive neural networks over pixels alone. Some tasks may do better with detailed joint angle representations, while this small behavior classification task can use the bounding box location and motion for classification in fewer dimensions.

**Table 4:** Table of the classification accuracy for each input representation on a held-out set of 400 test video clips for six behaviors: “standing/background”, “sleeping”, “wheel running”, “walking/running through the cage”, “grooming/scratching”, and “eating”.

Feature	acc (Behavior)
Images	56.8
2D boxes	**59.3**
2D points	53.6
3D points	54.8
3D angles	44.5

Best result is in bold.

## Conclusions

Here, we present a method that infers the 3D pose of mice from single view videos, describing each component of our analytical pipeline and its overall performance. We evaluated the performance of our method in terms of the accuracy of the primary output: keypoints (e.g. Table 2). However, 3D keypoints are not meaningful phenotypes by themselves, so we evaluated the ability of these outputs to capture biologically-relevant changes in mouse behavior. For two biological perturbations that are known to affect gait (age and mutation of Eif2B), the outputs from multiple stages of our method (bounding boxes, 2D keypoints, 3D keypoints, and 3D joint angles) were able to predict biological status (Table 3). Importantly, there was little advantage in converting 2D keypoints to 3D keypoints, but there was considerable advantage in converting 3D keypoints to 3D joint angles. Beyond demonstrating the efficacy of our particular method, this result added insight into what aspect of pose data can best capture biology. We demonstrate that the 3D joint angles enable predicting health related attributes of mice more easily than other features.

Our method offers compelling opportunities for continuous, non-invasive monitoring. In addition to the utility of pose estimates as consolidated inputs for the black-box classification of biological attributes, our system also provides an alternative solution to custom hardware for determining gait parameters such as stride length (Fig. 5). Future work includes improving the accuracy of the 3D pose and extending this method to animal social interactions.

The ML models in our pipeline were trained and evaluated across videos of mice in a limited diversity of visual contexts. Though potentially robust in new environments, these models may require retraining with additional data matching new visual environments in some cases. To enable the extension of our approach, or similar approaches, we provide images of single mice with annotated 2D keypoints; labelled videos of multi-mouse tracking; and anatomical CT scans used to construct our shape prior (“Data availability” Section). We hope this Mouse Pose Analysis Dataset and the accompanying models and code will serve as a valuable community resource to enable new research.
